# Implicating Calpain in Tau-Mediated Toxicity *In Vivo*

**DOI:** 10.1371/journal.pone.0023865

**Published:** 2011-08-17

**Authors:** James B. Reinecke, Sarah L. DeVos, James P. McGrath, Amanda M. Shepard, Dustin K. Goncharoff, Don N. Tait, Samantha R. Fleming, Michael P. Vincent, Michelle L. Steinhilb

**Affiliations:** Department of Biology, Central Michigan University, Mount Pleasant, Michigan, United States of America; Thomas Jefferson University, United States of America

## Abstract

Alzheimer's disease and other related neurodegenerative disorders known as tauopathies are characterized by the accumulation of abnormally phosphorylated and aggregated forms of the microtubule-associated protein tau. Several laboratories have identified a 17 kD proteolytic fragment of tau in degenerating neurons and in numerous cell culture models that is generated by calpain cleavage and speculated to contribute to tau toxicity. In the current study, we employed a *Drosophila* tauopathy model to investigate the importance of calpain-mediated tau proteolysis in contributing to tau neurotoxicity in an animal model of human neurodegenerative disease. We found that mutations that disrupted endogenous calpainA or calpainB activity in transgenic flies suppressed tau toxicity. Expression of a calpain-resistant form of tau in *Drosophila* revealed that mutating the putative calpain cleavage sites that produce the 17 kD fragment was sufficient to abrogate tau toxicity *in vivo*. Furthermore, we found significant toxicity in the fly retina associated with expression of only the 17 kD tau fragment. Collectively, our data implicate calpain-mediated proteolysis of tau as an important pathway mediating tau neurotoxicity *in vivo*.

## Introduction

Alzheimer's disease (AD) belongs to a group of related neurodegenerative disorders known as tauopathies, whose common pathological feature involves the deposition of abnormal, aggregated, highly phosphorylated tau in neuronal cells. Hyperphosphorylated tau is the major component of protein aggregates such as paired helical filaments (PHFs) and neurofibrillary tangles (NFTs) found in AD brain. Because mutations in the tau gene locus cause familial neurodegeneration with neurofibrillary tangle formation [1], [2], [3], [4], it is clear that primary abnormalities in tau are sufficient to cause neurodegeneration. However, the mechanisms leading to tau-induced neuronal cell death still require further definition.

In order to study the role of tau in disease, several animal model systems have recently been developed. In 2001, Wittmann and colleagues created a tauopathy model using the fruit fly *Drosophila melanogaster*. Transgenic flies expressing wild-type and mutant forms of human tau recapitulate many features of the human disease including adult onset, progressive neurodegeneration, early death, enhanced toxicity of mutant tau, and accumulation of abnormal tau [5]. The tau-induced rough eye phenotype provides an excellent model system to evaluate tau toxicity: genetic modifiers and pharmacological agents are capable of enhancing and suppressing the tau rough eye [6].

The role that kinases play in mediating neurodegeneration in Alzheimer's disease and related disorders has been a topic of significant interest. One potential manner in which phosphorylation may affect tau toxicity is by influencing the susceptibility of tau to proteolysis. Tau is known to be the target of several intracellular proteases including caspases, cathepsins, the proteasome, and calpain [7], [8], [9]. Importantly, tau fragments have recently been reported to be released from degenerating cultured rat cortical neurons, and small tau fragments are being assessed for their utility as biomarkers in preclinical and clinical assessment of acute neurodegenerative disorders [10]. The generation of a 17 kD tau fragment has been reported in cerebellar granule cells undergoing apoptosis [11] as well as hippocampal neurons exposed to aggregated Aβ [12], [13]. The 17 kD fragment is likely generated by calpain cleavage of tau and the 17 kD fragment itself is found to be toxic when exogenously expressed in neuronal and non-neuronal cells [12]. Most recently, Ferreira and Bigio [14] reported elevated levels of the 17 kD tau fragment and enhanced calpain activity in the brains of patients with AD and other tauopathies. These data implicate calpain-mediated proteolysis of tau as an important pathway mediating tau toxicity.

Calpain targets several proteins, including notably the structural components of the cytoskeleton such as MAP-1, MAP-2, actin, spectrin, and neurofilaments and has been shown to cleave tau directly in PC12 cells [15], [16]. In addition, calpain catalyzes the p35→p25 event that activates cyclin-dependent kinase 5 (cdk5), a proline-dependent kinase implicated in tau hyperphosphorylation in AD and other tauopathies [17]. Recently, calpain has been shown to participate in processing of amyloid precursor protein by increasing levels of BACE-1, leading to increased Aβ production, and ultimately elevated plaque load [18].

Calpain is a calcium-activated intracellular cysteine protease found in organisms ranging from yeast to humans. In humans, there are 2 types of calpain, classified based on the concentration of calcium (Ca^2+^) required to elicit enzymatic activity. Calpain I (µ-calpain) is activated at micromolar Ca^2+^ concentrations and is the major form expressed in neuronal cells. Calpain II (m-calpain) is active when cellular Ca^2+^ concentrations are in the millimolar range [19]. Although mammals possess at least 14 calpain genes, *Drosophila* has only four (CalpainA-D) and only calpA and calpB are predicted to have enzymatic activity [20].

In addition to Alzheimer's disease, calpain has been implicated in the pathogenesis of other neurodegenerative diseases. Huntington's disease is caused by a polyglutamine (polyQ) tract expansion near the amino-terminus of the protein huntingtin. Mutation of two calpain cleavage sites in huntingtin renders the polyQ expansion less susceptible to proteolysis and aggregation, resulting in decreased toxicity in a cell culture model. In addition, a number of calpain family members appear to be increased and activated in Huntington's disease tissue culture and transgenic mouse models [21], [22]. A recent report examining proteolytic processing and disease-linked aggregation in Parkinson's disease found that calpain cleaves α-synuclein, leading to the formation of aggregated high-molecular weight species and adoption of β-sheet structure [23]. Dufty and colleagues detected calpain-cleaved α-synuclein in mouse and fly models of Parkinson's disease, as well as in the substantia nigra of human Parkinson's disease brain tissue.

Recently, there has been a significant increase in the development of cell culture model systems to study tau toxicity. In culture, the greatest challenge has been finding a cell line and isoform of tau that recapitulates the clinical features of tau in human disease, including aggregation, hyperphosphorylation and proteolytic degradation. Several useful cell models now exist using various strategies for evaluating tau toxicity. Canu *et al.* has effectively demonstrated the use of cerebellar granule cells undergoing apoptosis to study the effect of cell death on tau and microtubules [11]. SH-SY5Y neuroblastoma cells stably over-expressing tau have been used to evaluate tau phosphorylation and proteolytic degradation [8], [24]. To study the generation of 17kD tau proteolytic fragments, other groups have treated rat hippocampal neurons with pre-aggregated Aβ [12], [13], [14]. Inducible expression of the repeat domain of tau in the neuroblastoma cell line N2a recapitulates robust tau aggregation and formation of Alzheimer's-like paired helical filaments [25], [26]. In non-neuronal cell culture, human epithelial kidney (HEK293) cells expressing full-length tau have been treated with Congo red (a small-molecule agonist of tau aggregation) to study tau aggregation and the importance of phosphorylation [27]. Full-length tau and tau fragments have been expressed in Chinese hamster ovary (CHO) cells [12], [28]. These models set a precedent for the effective use of cell culture models to study tau toxicity.

Because the appearance of truncated tau fragments has profound significance in human disease, it is important to understand the effect of tau proteolysis not only in cell culture, but also in an intact animal system. The tauopathy model in *Drosophila* offers a unique system to analyze the role of calpain in tau-induced neurotoxicity: using the powerful genetic and molecular tools available in flies, we can assess the pathological importance of calpain cleavage of tau in an intact animal model of human neurodegenerative disease.

## Results

### Tau and Calpain Colocalize in *Drosophila* Neurons

Since we hypothesized that calpain cleavage of tau may be an important event in tau toxicity, we sought to determine whether tau and calpain possess overlapping localization in neurons. Although there are at least 14 human calpain-like protease domain-containing genes, flies have only four (calpA-D). Only calpA and calpB are predicted to have enzymatic activity. CalpC has been speculated to be the *Drosophila* equivalent of calpastatin, the endogenous mammalian inhibitor of calpain and calpD (originally called SOL for small optic lobe) is an atypical member of the calpain family that does not possess protease activity [20], [29]. As a consequence, we focused specifically on calpA and calpB.

**Figure 1:**
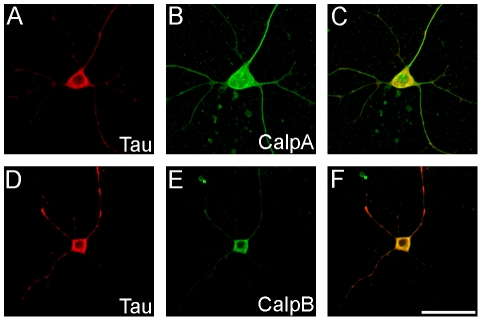
Colocalization of tau and calpain in *Drosophila* neurons. Neuronal cells cultured from late 3^rd^ instar larvae expressing tau^WT^ were stained for tau in red (A and D), calpain A and calpain B in green (B and E, respectively), with colocalization shown in yellow (C and F). The expression pattern of calpain A and B was similar to that of tau, with the primary site of colocalization in the neuronal cell body. Scale bar  = 5 µM.

To determine the subcellular localization of tau and calpain in *Drosophila* neurons, we used the *elav-GAL4* driver to express human tau (tau^WT^) in all post-mitotic neurons. Neurons were isolated from *elav; tau^WT^* third instar larvae/white pre-pupae and stained for human tau and endogenous fly calpains. As shown in Figure 1, tau displayed perinuclear staining with some staining shown in the processes radiating out from the cell body (panels A and D). CalpA appears to be ubiquitously expressed throughout the cell body and projections (panel B) while calpB expression was primarily localized to the soma in a perinuclear pattern (panel E). Both calpA and calpB showed colocalization with tau (panels C and E), with the site of greatest overlapping subcellular distribution in the cell body.

Because calpA and calpB are calcium-activated proteases, we tested whether stimulation with ionomycin impacted the colocalization of tau with calpain. We found no difference in localization between ionomycin-treated and untreated neurons (data not shown).

### Calpain Mutants Suppress Tau-induced Toxicity in the Fly Eye

In order to assess the pathological significance of calpain cleavage of tau in an intact animal, we utilized a *Drosophila* tauopathy model. This model is particularly attractive as the rough eye phenotype of human tau-expressing flies can be enhanced and suppressed by genetic modifiers [6]. We genetically modulated calpain in flies using mutant alleles of endogenous *Drosophila* calpain to decrease expression levels. For both calpA and calpB we selected one deficiency line and two P-element insertion lines for analysis.

**Figure 2:**
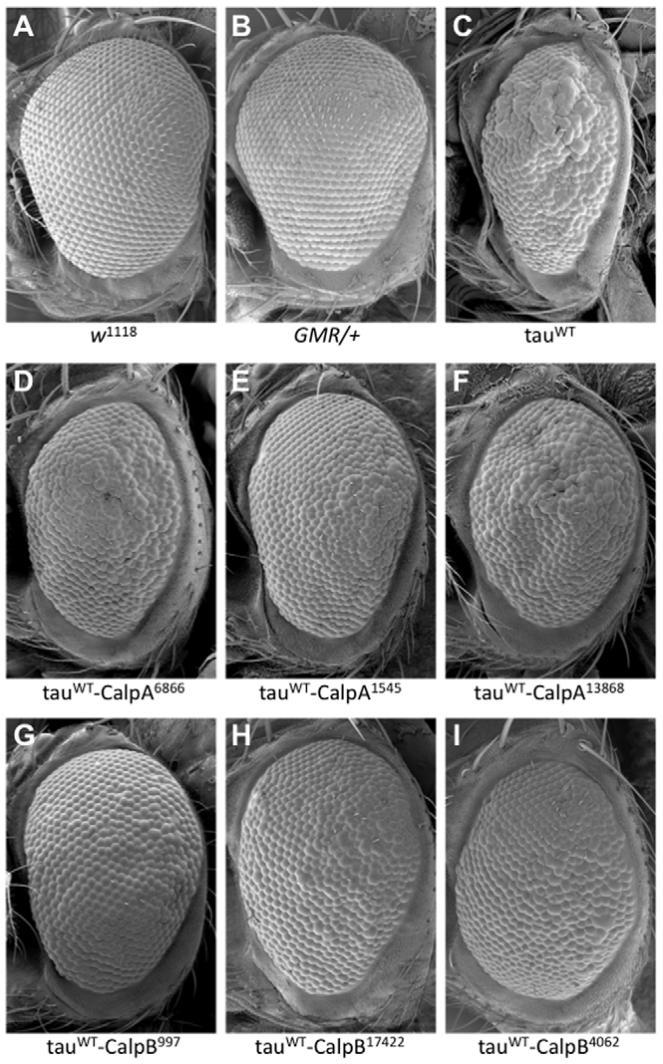
Calpain mutants suppress tau-induced toxicity in the fly eye. As shown using scanning electron microscopy, compared to the normal external appearance of control flies (*w^1118^* in A; *GMR-GAL4/+* in B), flies expressing wild-type human tau (tau^WT^; C) displayed the moderately rough eye phenotype characterisitc of tau toxicity. Genetic disruption of calpain A (D–F) or calpain B (G–I) using either deficiency lines (D and G) or P-element insertions (E–F and H–I) effectively suppressed tau toxicity.

As shown in Figure 2 using scanning electron microscopy, non-transgenic flies (Figure 2A) and flies possessing only the retinal driver *GMR-GAL4* (Figure 2B) possess an ordered ommatidial morphology characterized by a regular array of bristles and lenses. Expression of tau^WT^ in the retina caused a moderate rough eye phenotype characterized by disruption of the ordered ommatidial arrangement and a moderate reduction in the size of the eye (Figure 2C). Compared to tau^WT^-expressing flies, transgenic flies expressing tau^WT^ together with calpA^6866^, calpA^1545^, or calpA^13868^ had reduced tau toxicity as shown by suppression of the rough eye phenotype (Figure 2D–F). Tau toxicity was also modestly reduced in flies expressing tau^WT^ together with any of the calpB-reducing alleles calpB^997^, calpB^17422^ or calpB^4062^ (Figure 2G–I). Collectively, these results suggest that genetically reducing calpA or calpB is sufficient to suppress tau toxicity *in vivo*.

**Figure 3:**
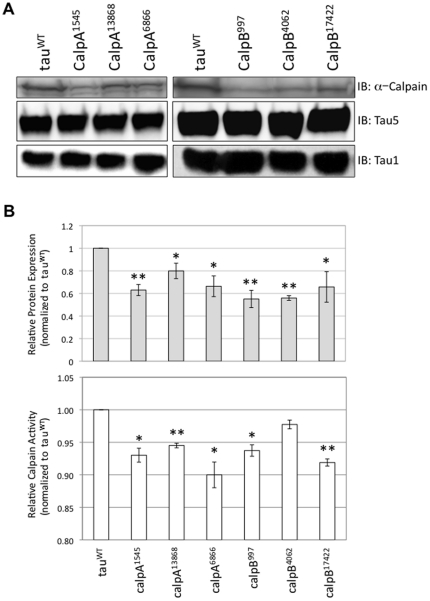
Calpain protein expression level and enzyme activity are decreased in calpain mutants. A. Using antibodies specific to calpain A or calpain B, western blot analysis showed that compared to tau^WT^-expressing flies, all of the calpain mutant lines have lower levels of calpain expression (*top*). Tau expression levels for all of the calpain mutants are equivalent to the expression level of tau in tau^WT^-expressing flies using both Tau 5 and Tau-1 antibodies (*bottom two panels*). B. When normalized to the level of calpain in tau^WT^-expressing flies, densitometric analysis revealed reduced calpain expression in all calpain mutant lines tested (*top*). The values shown represent the mean of three or more independent experiments. When normalized to calpain activity in tau^WT^-expressing flies, all calpA and calpB mutants have reduced calpain activity (*bottom*). The values shown represent the mean of enzyme activity measured in triplicate. Error bars for both graphs represent the standard error of the mean and data points indicated with asterisks are significant. *P<0.05, **P<0.01 (unpaired student t-test).

Having found that transgenic flies with mutations predicted to disrupt endogenous calpA or calpB suppressed tau toxicity, we verified that these flies indeed had reduced levels of calpain expression and calpain activity. As shown in Figure 3, quantitative western blot analysis confirmed that calpain expression levels were significantly reduced in all of the calpA and calpB mutants used. Compared to tau^WT^, calpain levels were reduced in the six calpain mutants by 20–45%. Importantly, tau expression levels in the calpain mutant lines were the same as in tau^WT^-expressing flies. Using a fluorogenic calpain activity assay, we also found that calpain activity was reduced in all of the calpA and calpB mutants tested.

### Creation of Tau^CR^ and Tau^17kD^

**Figure 4:**
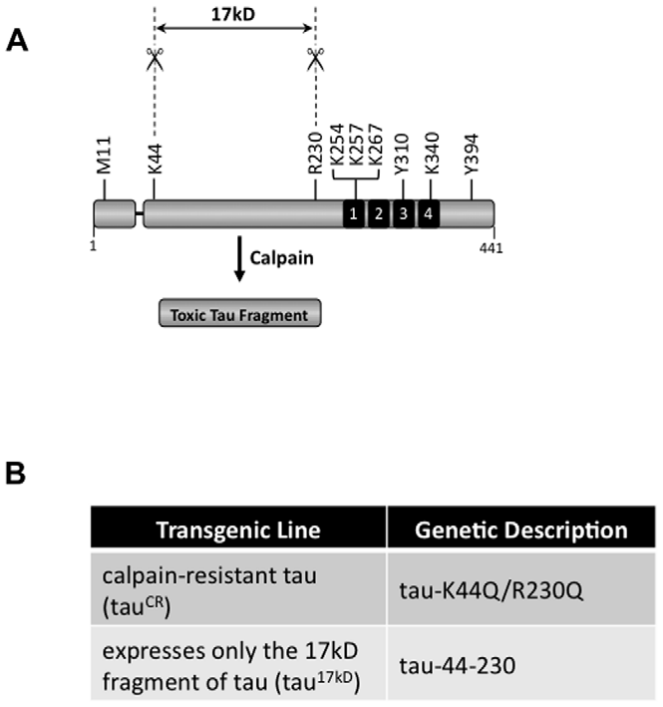
Cleavage of tau by calpain creates the 17 kD fragment. A. Schematic of 0N4R tau with the nine putative calpain cleave sites indicated. Proteolysis at lysine 44 (K44) and arginine 230 (R230) yields a 17 kD fragment of tau. Abbreviations: methionine (M), lysine (K), arginine (R), and tyrosine (Y). B. The K44Q/R230Q mutation is predicted to disrupt the recognition motif for calpain at K44 and R230 and thus creates a calpain-resistant (tau^CR^) form of tau for expression in *Drosophila*. PCR was used to create a second tau mutant (tau^17kD^) in which only the amino acids corresponding to the reputed toxic 17 kD fragment (aa 44-230) are expressed in *Drosophila*.

Based on antibody analysis by other researchers [11], [30] and the predicted calpain cleavage sites in tau [31], [32], the reputed toxic 17 kD tau fragment is likely to include amino acids 45–230. Calpain favors cleavage at methionine, alanine, arginine, or lysine preceded by leucine or phenylalanine [31], [32]. Full-length human tau contains 9 putative calpain cleavage sites: Met11, **Lys44**, **Arg230**, Lys254, Lys257, Lys267, Tyr310, Lys340, and Tyr 394 (17 kD fragment start/end shown in bold; see Figure 4) [30]. To determine whether inhibiting calpain cleavage of tau could modify neurotoxicity, we generated a calpain-resistant tau construct (tau^CR^) for expression in *Drosophila* in which calpain cleavage sites at K44 and R230 were mutated to glutamine (Q) using site-directed mutagenesis (tau-K44Q/R230Q; see Figure 4). Glutamine was chosen as a substitution since it is structurally similar to lysine and arginine but it no longer has a charged polar side chain. Since research from other laboratories suggested that the 17 kD fragment of tau has intrinsic toxicity in neuronal and non-neuronal cell types [12], [33], [34], [35], we used PCR to create an additional tau mutant (tau^17kD^) in which only the amino acids corresponding to the reputed toxic 17 kD fragment (aa 44–230) were expressed in *Drosophila* (Figure 4).

**Figure 5:**
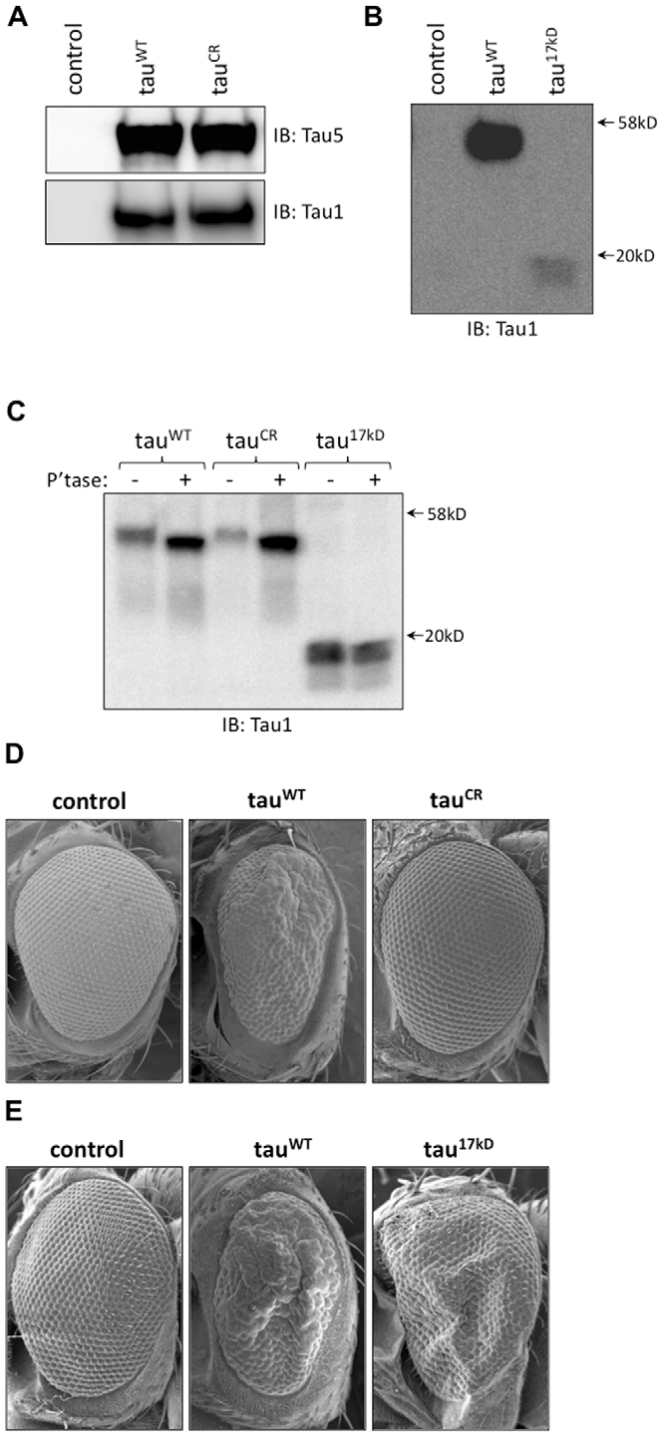
Modifying two putative calpain cleavage sites in tau significantly impacts tau toxicity *in vivo*. A. Western blot analysis of fly head homogenates revealed equivalent tau expression levels in tau^CR^-expressing flies compared to tau^WT^-expressing flies. There is no cross-reactivity of the Tau-1 or Tau 5 antibody with homogenates from control flies (*GMR/+*). B. Expression of the 17 kD fragment could not be detected at 25°C and instead required crosses to be conducted at 31°C. Possessing only the coding sequence for amino acids 44-230, tau^17kD^-expressing flies made only a ∼17 kD tau fragment that is found at lower abundance than that of full-length tau found in tau^WT^–expressing fly head homogenates with equivalent protein loading. C. Treatment of fly head homogenates from tau^WT^-, tau^CR^- and tau^17kD^-expressing flies with lambda phosphatase indicated that both the wild-type and calpain-resistant forms of tau experienced a mobility shift with phosphatase treatment whereas the 17 kD fragment was resistant to phosphatase treatment. To ensure we would visualize a potential molecular weight shift of the 17 kD fragment by western blot, the phosphatase assay employed three times more fly head homogenate from tau^17kD^-expressing flies than tau^WT^ or tau^CR^ flies. D. When compared to the rough eye phenotype in tau^WT^-expressing flies, tau^CR^ flies demonstrated no detectable tau toxicity. E. At 31°C, transgenic flies that express only the 17 kD tau fragment (tau^17kD^) showed equivalent or slightly enhanced tau toxicity compared to the toxicity associated with expressing tau^WT^ in the retina, suggesting that the 17 kD fragment possesses intrinsic toxicity *in vivo*. Control flies in D and E are *GMR/+*.

We used western blot analysis to assess tau expression levels in our new tau^CR^ and tau^17kD^ transgenic lines. As shown in Figure 5A, tau expression levels are identical in tau^WT^- and tau^CR^-expressing flies. Interestingly, expression of the 17 kD fragment in flies was more challenging. We received only five transgenic lines from the injection service we contracted and the lines we recovered expressed low amounts of 17 kD tau. Initial analyses with these five lines showed that when expressed with *GMR-GAL4* at 25°C, there was no detectible modification of the non-transgenic eye phenotype and concurrently, we were unable to detect tau expression in these flies. To enhance expression of the 17 kD fragment, we set-up crosses with tau^17kD^- and tau^WT^-expressing flies reared at 31°C. We found that with equivalent protein loading, tau^17kD^-expressing flies produce a 17 kD form of tau that was recognized by the Tau-1 antibody (Figure 5B). We found that the 17 kD tau fragment was lower in abundance than full-length tau^WT^ in *Drosophila*. However, given that the reputed toxic 17 kD fragment is a cleavage product of tau, we did not bias ourselves in thinking that expression levels of tau^17kD^ should be equivalent to that of tau^WT^. Since tau^17kD^ flies do not possess the full-length human tau transgene, as expected, we did not see full-length tau in these flies.

To determine whether the proteins generated by our tau^CR^- and tau^17kD^-expressing flies were phosphorylated *in vivo*, we performed a phosphatase assay. As shown in Figure 5C, phosphatase treatment reduced the molecular weight of both wild-type and calpain-resistant tau, but did not influence the mobility of the 17 kD tau fragment. The mobility shift observed with phosphatase treatment supports that tau^CR^ is synthesized as a full-length protein and is indeed phosphorylated to the same extent as tau^WT^ *in vivo*. The resistance of the 17 kD fragment to phosphatase treatment suggests that the 17 kD tau fragment is not phosphorylated at sites that contribute significantly to a gel mobility shift.

### Tau^CR^ and Tau^17kD^ Significantly Modify Tau Toxicity *in vivo*

To determine if preventing cleavage of tau at the putative calpain cleavage sites of K44 and R230 reduced the neurotoxicity of tau in an intact animal model, we assessed expression of tau^WT^ and tau^CR^ in the *Drosophila* retina using the *GMR-GAL4* driver. As shown in Figure 5D, compared to the rough eye phenotype seen in tau^WT^-expressing flies, tau^CR^-expressing flies have markedly reduced tau toxicity. In fact, the reduction in toxicity is so significant that aside from a slight bristle defect, these flies are nearly indistinguishable from non-transgenic flies. Thus, mutation of K44 and R230 was sufficient to abrogate tau neurotoxicity *in vivo*.

To establish whether expression of the 17 kD fragment of tau was toxic in an intact animal model, we used the *GMR-GAL4* driver to direct expression of the reputed toxic tau fragment in the fly eye. At 31°C, tau^17kD^-expressing flies possessed a rough eye phenotype with equivalent toxicity compared to tau^WT^-expressing flies also crossed at 31°C (Figure 5E). Since there is equivalent toxicity in tau^WT^- and tau^17kD^-expressing flies despite much lower levels of 17 kD expression (see Figure 5B), these results are consistent with substantially increased toxicity of the 17 kD fragment of tau. Collectively, these results supported our hypothesis that calpain is involved tau-mediated toxicity *in vivo*.

## Discussion

Calpain proteolysis is emerging as an important mediator of neurodegenerative disease. Recently, calpain has been implicated in the production of toxic fragments in two other diseases besides Alzheimer's disease: α-synuclein in Parkinson's disease [23] and huntingtin in Huntington's Disease [21], [22], [36]. All share a common theme: calpain-cleaved, truncated forms of tau, α-synuclein, and huntingtin have pathologic significance in their respective diseases. It is therefore of the utmost importance to understand at the molecular level the relationship between calpain and the generation of toxic protein fragments. We are in a unique position to study calpain cleavage of tau using an intact animal model of human neurodegenerative disease, the *Drosophila* tauopathy model.

In humans, calpain activity increases during normal aging, however individuals with familial AD and other tauopathies have significantly elevated levels of active calpain [37], [38] as well as the 17 kD fragment of tau [14]. Activated calpain is detected prior to tau abnormalities and neuronal cell death, suggesting that calpain activation is not a consequence of the neurodegenerative process, but rather precedes and promotes neurodegeneration [19]. One study in particular found that the active form of calpain was present together with hyperphosphorylated tau in 50–75% of preparations from several human disorders, including tau pathology associated with Alzheimer's disease, Down's syndrome, diffuse Lewy body disease, progressive supranuclear palsy, and corticobasal degeneration [37]. To test whether tau and calpain exist in the same subcellular compartment in neuronal cells in our model system, we prepared primary cultures of *Drosophila* neurons for confocal analysis. As shown in Figure 1, human tau colocalizes with both calpA and calpB in *Drosophila* neurons. This places tau and calpain in close proximity to execute the cleavage event to produce potentially toxic tau fragments in our model. Our observation was consistent with other reports that find activated calpain in association with hyperphosphorylated tau [37], [39].

Having first established that tau and calpain colocalize in the *Drosophila* nervous system, we examined whether altering calpain expression genetically could modify the tau-induced rough eye phenotype observed in the *Drosophila* tauopathy model. We hypothesized that if calpain plays a role in eliciting tau toxicity, genetically decreasing the amount of calpain in flies may suppress tau toxicity. Using fly stocks available from public stock centers we found exactly this result: calpain mutant lines that function to decrease physiological levels of calpain suppressed the tau rough eye phenotype *in vivo* (Figure 2). Western blot analysis confirmed that the suppression of the tau-induced rough eye was not a consequence of modifying tau levels in these flies since tau expression levels found in fly head homogenates were unchanged compared to flies expressing only tau^WT^. Compared to tau^WT^-expressing flies, calpain expression levels, however, were significantly reduced in homogenates from flies expressing any of the calpA or calpB mutants together with tau^WT^ (see Figure 3). In addition to assessing calpain expression levels by western blot, we employed quantitative RT-PCR as a secondary measurement of calpain expression level and found >2 fold reduction in calpA mRNA in the calpA mutants compared to tau^WT^ and >1.1 fold reduction in calpB mutants (data not shown). Using a fluorogenic calpain activity assay, we observed decreased calpain activity in all of the calpA and calpB mutants. Although we found a 20-45% decrease in calpain expression levels, we observed a ≤10% reduction in calpain activity. It is important to note that while the quantitative protein expression data were generated using antibodies that recognize only calpA or calpB, the calpain activity assay measures the contribution of both calpA and calpB to total enzyme activity. Since the genetic reduction of either calpA or calpB affects only one of the two functional calpain genes, it is not surprising that the reduction in calpain expression level is not identical to the overall reduction in calpain enzymatic activity. Given that tau toxicity is suppressed under conditions where calpain levels are genetically reduced, we are currently making calpain over-expressing transgenic lines to test whether elevated levels of calpain enhance tau toxicity.

Inhibiting calpain pharmacologically or with overexpression of calpastatin (the endogenous inhibitor of calpain) has been documented to impact tau proteolysis [11], [18], [40], [41], [42] or reduce neuronal death [43], [44]. Using the calpain inhibitor MDL 28,170, we established a dose-response curve and found the optimum drug concentration for our feeding experiments to be 1 µM. However, upon drug administration we found no effect of MDL 28,170 on tau toxicity (data not shown).

Although compelling, the modifier analysis does not distinguish whether the impact of calpain upon tau is direct or indirect. Since calpain is involved with activation of cdk5, a major tau kinase, several laboratories have suggested that the role of calpain in disease may be to promote tau hyperphosphorylation, ultimately causing cytoskeletal disruption and neuronal apoptosis [17], [45], [46]. Other researchers postulate that as a protease, calpain interacts with hyperphosphorylated tau in an effort to degrade the protein to avoid cellular toxicity [13], [14], [47]. To determine whether calpain promotes tau toxicity by cleaving tau directly, we made specific mutations to alter the putative calpain cleavage sites on tau that are predicted to produce the toxic 17 kD fragment. Significant effort has been made to discern the amino acid consensus sequence for calpain cleavage of target proteins. Given the wide range of calpain targets, no single consensus sequence motif has prevailed. Rather, higher-order structural features such as peptide bond accessibility, backbone conformation, and three-dimensional structure were found to be important. In general however, calpain favors cleavage at methionine, alanine, arginine, or lysine preceded by leucine or phenylalanine [31], [32]. As shown in Figure 4A, tau contains 9 putative calpain cleavage sites [30]. Analysis of the 17 kD tau fragment using sequence-specific monoclonal antibodies suggests that that lysine 44 (K44) and arginine 230 (R230) define the boundaries of the fragment generated by calpain cleavage that correlates with toxicity [11], [12]. Since the interaction of the P1 residue of a substrate with the S1 subsite of the enzyme is critical for substrate orientation leading to efficient catalysis, we elected to mutate the P1 residues at the two critical calpain cleavage sites, namely K44 and R230. Glutamine was selected as a substitution for K44 and R230 since it is structurally most similar to lysine and arginine but it no longer has a charged polar side chain. As shown in Figure 5, with equivalent protein expression levels, mutation of only two amino acids in tau at the predicted sites of calpain cleavage was sufficient to abrogate tau toxicity *in vivo*.

At least two other groups have attempted to block production of the 17 kD fragment using site-directed mutagenesis at the predicted calpain cleavage sites of tau using the P_2_-P_1_ rule [32], which predicts the preferred residues surrounding the scissile bond for calpain cleavage of target proteins [12], [28]. While Park and Ferreira found that L43A/V229A prevented the appearance of the 17 kD fragment, Garg *et al.* found that neither L43A alone nor L43A together with V229A prevented generation of the 17 kD fragment of tau. While our results are consistent with Park and Ferreira whose experiments utilized cultured neurons, we speculate that the different results obtained by Garg and colleagues may be due, at least in part, to the fact that their experiments were performed on constructs expressed in non-neuronal Chinese Hamster Ovary (CHO) cells using recombinant calpain 2.

The generation of a 17 kD tau fragment has been reported in cerebellar granule cells undergoing apoptosis [11], [47], [48] and hippocampal neurons exposed to aggregated Aβ [12], [13], [33], [35], and was found to be toxic when exogenously expressed in neuronal and non-neuronal cells [12]. Most recently, Ferreira and Bigio [14] reported increased levels of the 17 kD tau fragment and elevated calpain activity in brain samples from patients with tauopathies. We were interested in determining if the intrinsic toxicity of the 17 kD fragment can be extended beyond cell culture models to establish whether the 17 kD tau fragment elicited toxicity in an intact animal system as well. We found that the 17 kD fragment possessed intrinsic toxicity *in vivo* since expression of even modest levels of the 17 kD fragment resulted in substantial toxicity in the fly retina (Figure 5E).

In *Drosophila*, several molecular pathways have been implicated in tau neurotoxicity and influence neurotoxicity in the fly eye at different times in development. While the unfolded protein response protects against tau neurotoxicity [49], lysosomal dysfunction [50], [51], abnormal bundling and accumulation of F-actin [52] and other cytoskeletal proteins [53], [54], oxidative stress [55], and tau phosphorylation [56], [57], [58], [59] are among the pathways shown to promote tau neurotoxicity *in vivo*. With respect to the rough eye phenotype in tau^17kD^-expressing flies, we are not certain of the mechanism controlling the tau-induced neurotoxicity we observe.

We have been largely unsuccessful at visualizing the 17 kD fragment in tau^WT^-expressing *Drosophila* lines unless we are specifically expressing this fragment using our tau^17kD^ transgenic lines. Although we have visualized a very faint band corresponding to the 17 kD fragment in tau^WT^-expressing flies (unpublished observation), these experiments typically require homogenates prepared with >300 fly heads and the appearance of the 17 kD fragment is sporadic and typically at the very limit of detection using western blot analysis. We suspect that the 17 kD fragment may be quickly degraded in flies and therefore its presence is particularly difficult to monitor. Consistent with this finding, others using *in vitro* calpain cleavage assays can only detect the 17 kD fragment when tau is in a soluble form [14]. Given that in the fly tauopathy model, the altered conformation and hyperphosphorylated state of tau correlates with tau toxicity in the absence of neurofibrillary tangle formation [5], it is possible that the structure and/or phosphorylation status of tau may be important for generating the 17 kD fragment.

Several studies have suggested that the primed sites of substrates (i.e. amino acids C-terminal to the scissile bond) are significantly involved in peptide recognition and the kinetics of cleavage by calpain [31], [32], [60]. In an extensive search for calpain substrates within the Swiss-Prot and TrEMBL protein databases, Tompa *et al.* reported that the frequency of the (S/T)P dipeptide in the P1'-P2' position was discovered with a surprisingly high frequency (5–10X above average) among calpain substrates [32]. Interestingly, threonine^231^ (T^231^) and proline^232^ (P^232^) occupy the P1'-P2' position at the putative R^230^ calpain cleavage site of tau. T^231^ is among a group of 14 SP/TP motifs present in tau and has been shown to be hyperphosphorylated in Alzheimer's disease and other tauopathies [61], [62] [57]. Phosphorylation at T^231^ (together with S^235^) creates the TG3 disease-associated phosphoepitope, a site phosphorylated by several kinases including cdk5, a proline-directed kinase that is activated by conversion of p35→p25 by calpain [17]. The presence of T^231^P^232^ at the P1'-P2' position would support a potential mechanism in which phosphorylation regulates the susceptibility of tau to calpain cleavage and the generation of potentially toxic proteolytic fragments.

## Materials and Methods

### Constructs, Genetics, and Stocks

All crosses were performed on standard cornmeal based medium. Fly stocks *UAS-tau^WT^*, GMR-GAL4 and elev-GAL4 were provided by Dr. Mel Feany and have been described previously [5], [57]. The following transgenic strains are described in Flybase (http://flybase.bio.indiana.edu/) and were purchased from Bloomington (http://fly.bio.indiana.edu/) or Exelixis (https://drosophila.med.harvard.edu/): CalpA^6866^ (Df(2R)BSC26, Bloomington); CalpA^1545^ (pBac{RB}CalpA[e01545], Exelixis); CalpA^13868^ (P{SUPorP}KG05080, Bloomington); CalpB^997^ (Df(3L)AC1, Bloomington); CalpB^17422^ (P{EPgy2}CalpB[EY17422], Bloomington); CalpB^4062^ (PBac{PB}CalpB[4062], Exelixis). Vector pBS-htau24 (Wittmann 2001) served as the template for generation of both calpain-resistant tau (tau^CR^; tau-K44Q/R230Q) and the 17kD fragment of tau (tau^17kD^; tau^44-230^) using the QuikChange site-directed mutagenesis kit (Stratagene, La Jolla, CA). For tau^CR^ the forward primer for K44Q was 5′CGGACGCTGGCCTG**CAA**GCTGAAG-AAGCAGG 3′ and the reverse primers was 5′ CCTGCTTCTTCAGC**TTG**CAGGCCAG-CGTCCG 3′. pBS-htau^K44Q^ was used as the template for the R230Q mutation to make tau^K44Q/R230Q^ using the forward primer 5′ GGTGGCAGTGGTC**CAG**ACTCCACCCAA-GTCG 3′ and the reverse primer 5′ CGACTTGGGTGGAGT**CTG**GACCACTGCCACC 3′. For tau^17kD^ the forward primer was 5′ GATCGCGGCCGCATGAAAGCTGAAGAAGCAGGC 3′ and the reverse primer was 5′ GATCCTCGAGTCACAGATCCTCTTCTGAGATGAGTTTTTGTTCA-CGGACCACTGCCACCT 3′. The tau^CR^ and tau^17kD^ constructs were subcloned into the pUAST vector and sequenced to verify the authenticity of each mutant. Mapped and balanced lines were created using the *Drosophila* embryo injection services of The Best Gene, Inc. (Chino Hills, CA). For both tau^CR^ and tau^17kD^, we analyzed 5–8 mapped and balanced transgenic lines and used quantitative western blot analysis to determine protein expression level.

### *Drosophila* Primary Cell Culture

Primary neurons were derived as described previously [63]. Briefly, CNS from surface sterilized (1 minute in 70% ethanol) late wandering third instar larvae, pre-pupae (0–5 hours), or 50% pupae were dissected in sterile calcium free buffer (800 mg NaCl, 20 mg KCl, 5 mg NaH_2_PO_4_, 100 mg NaHCO_3_, and 100 mg glucose in 100 mL of culture grade water). Dissected brains were placed in a solution containing 0.2 mg/mL collagenase (Type I, Sigma, St. Louis, MO) diluted in calcium free buffer for 30 minutes at room temperature. CNS were washed three times for 5 minutes in plating medium (Schneider 2 media (Invitrogen, Carlsbad, CA) plus 50 µg/mL insulin (Sigma) and 10% heat-inactivated normal bovine serum (Rockland Immunochemicals, Philadelphia, PA)). CNS were dispersed into single cells by repeated passage through a pipet tip (∼40 times) and plated (100 µL) onto UV-sterilized circular coverslips (Electron Microscopy Sciences, Hatfield, PA) that had been coated with 167 µg/mL concanavalin A (Sigma) and 1.67 µg/mL mouse-laminin (Invitrogen) for 2 hours at 37°C. Cells were allowed to attach to the coverslips for approximately 2–3 hours at 25°C before additional plating medium was added. Cells were grown at 25°C for 1–3 days prior to processing.

### Immunohistochemistry

For immunofluorescence studies of primary neurons, plating medium was removed and cells were rinsed two times in phosphate-buffered saline (PBS; Denville Scientific, Metuchen, NJ) followed by fixation in 4% paraformaldehyde (Electron Microscopy Sciences, Hatfield, PA)/PBS for 10 minutes at room temperature. After three washes in PBS plus 0.3% Triton-X 100 (PBT), cells were blocked for 30 minutes at room temperature in PBT plus 0.2% bovine serum albumin (HyClone, Logan, UT) and 5% normal goat serum (PBTB; Rockland Immunochemicals). Fixed cells were then incubated in the appropriate primary antibody overnight at 4°C: rat α-elav (1∶100; Developmental Studies Hybridoma Bank (DSHB, Iowa City, IA)), mouse α-22C10 (1∶20, DHSB), mouse α-Tau-5 (1∶100; DHSB), rabbit α-Calpain A (1∶1000; Friedrich and Farkas), and rabbit α-Calpain B (1∶1000; Friedrich and Farkas). Following primary antibody incubation, samples were washed three times (10 minutes) in PBT followed by incubation with the appropriate fluoro-conjugated secondary antibody (1∶200; Jackson ImmunoResearch, West Grove, PA) for one hour at room temperature. After three washes in PBT, the cells were mounted in Prolong Gold (Invitrogen) and visualized on an Olympus FluoView 300 Confocal Microscope with a 60X oil immersion lens at a 3x confocal zoom. z-stacks were made from three 0.5 µm slices.

### Scanning Electron Microscopy

Adult flies were fixed in 4% glutaraldehyde (Electron Microscopy Services) for ≥24 hours, dehydrated through a graded series of ethanol solutions, critically point dried (Polaron critical point dryer), and sputter coated (Denton Desk II sputter coater). Flies were imaged using a JEOL JSM-840A Scanning Electron Microscope.

### Protein Isolation, SDS-PAGE, Western Blot

Fly head homogenates were prepared in Laemmli buffer (Sigma) from adult flies 1-day post eclosion. Protein samples were boiled for 5 minutes, centrifuged for 5 minutes at 13,000*xg* to remove insoluble debris, and resolved by Sodium Dodecyl Sulfate Polyacrylamide Electrophoresis (SDS-PAGE) using 12% separating gels (Bio-Rad, Hercules, CA). Proteins were transferred to activated PVDF (Millipore, Billerica, MA), blocked in 5% milk in phosphate buffered saline (Denville Scientific) with 0.1% Tween-20 (Fisher Scientific, Pittsburg, PA), and incubated with one of the following primary antibodies at 4°C overnight: rabbit α-calpain A (1∶10,000; Friedrich and Farkas), rabbit α-calpain B (1∶10,000; Friedrich and Farkas), Tau 5 (1∶1000; Developmental Studies Hybridoma Bank) or mouse α-Tau1 (1∶5,000; Millipore). Membranes were washed with PBST prior to incubation with the appropriate α-mouse or α-rabbit horseradish peroxidase (HRP) conjugated secondary antibody (1∶10,000) followed by chemiluminescent signal detection with the SuperSignal West Pico Detection Kit (Pierce, Rockford, IL). The Kodak GelLogic 2200 was used for digital image capture and densitometry analysis. Statistical analysis was performed using the student t-test with densitometry data from ≥3 separate experiments.

### Phosphatase Assay

Phosphatase treatment was performed as described previously [57]. Fly head homogenates from transgenic lines expressing tau^WT^, tau^CR^ and tau^17kD^ were prepared in 1X lambda phosphatase buffer (New England Biolabs, Beverly, MA) containing a protease inhibitor cocktail (Roche, Indianapolis, IN). To enhance visualization of the 17 kD fragment, homogenates for tau^17kD^ contained three times more total protein than tau^WT^ or tau^CR^. Phosphatase-treated samples were incubated with lambda protein phosphatase at 37°C for 3 hours. After the addition of Laemmli buffer (Sigma), homogenates were subjected to SDS-PAGE and western blot analysis as described above.

### Calpain Activity Assay

Calpain activity was measured using a fluorogenic activity assay kit following the protocol provided by the manufacturer (EMD Chemicals, Inc., Gibbstown, NJ) with some modifications. Briefly, fly head homogenates were prepared in CytoBuster Protein Extraction Reagent (EMD Chemicals, Inc.) from adult flies 1-day post eclosion, centrifuged for 1 minute at 13,000*xg* to remove insoluble debris, and incubated with the substrate ((DABCYL)-TPLKSPPPSPR-(EDANS)) in activation buffer with reducing agent for 30 minutes at room temperature in the dark. Fluorescence was measured using an excitation wavelength of 320 nm and an emission wavelength of 460 nm using a fluorescence plate reader. Protein concentration was determined using a Bradford-based microassay procedure (Bio-Rad). Statistical analysis was performed using the student t-test with samples run in triplicate.
